# Membrane Insertion of MoS_2_ Nanosheets: Fresh *vs.* Aged

**DOI:** 10.3389/fchem.2021.706917

**Published:** 2021-06-25

**Authors:** Rui Ye, Wei Song, Xinwen Ou, Zonglin Gu, Dong Zhang

**Affiliations:** ^1^ Department of Physics, Zhejiang University, Hangzhou, China; ^2^ Institute of Quantitative Biology, Zhejiang University, Hangzhou, China; ^3^ College of Physical Science and Technology, Yangzhou University, Yangzhou, China; ^4^ College of Life Sciences, Zhejiang University, Hangzhou, China

**Keywords:** MD simulation, MoS_2_ nanosheet, lipid membrane, surface aging, insertion free energy

## Abstract

Fresh two-dimensional molybdenum disulfide (MoS_2_) absorbs the hydrocarbon contaminations in the ambient air and makes surface aging. To understand how the surface aging influences the interactions between MoS_2_ and biomolecules is important in the biomedical applications. Here, employing all-atom molecular dynamics simulations, we investigated the interactions of the fresh and aged MoS_2_ nanosheets with the lipid membranes of different components. Our results demonstrate that both the fresh and aged MoS_2_ nanosheets can spontaneously insert into the bilayer membranes. However, the fresh MoS_2_ nanosheet displays significantly stronger interaction and then has a larger penetration depth than the aged counterpart, regardless of the lipid components. The calculations of potential mean forces through the umbrella sampling further confirm that the insertion of fresh MoS_2_ into the lipid membranes is more energetically favorable. Moreover, we found that the fresh MoS_2_ nanosheet can cause a larger damage to the integrity of lipid membranes than the aged one. This work provides insightful understandings of the surface-aging-dependent interactions of the MoS_2_ nanosheets with biomembranes, which could facilitate the design of novel MoS_2_-based nanodevices with advanced surface properties.

## Introduction

Molybdenum disulfide (MoS_2_) is a representative transition metal dichalcogenides and has been recently attracted a significant number of interests in the scientific community. Previous studies have shown that the MoS_2_ nanosheets exhibit a wide range of applications in optoelectronics (Splendiani et al., 2010), field emission transistors (Radisavljevic et al., 2011), gas sensors (Qiu et al., 2012; Perkins et al., 2013), and hydrogen storage (Chen et al., 2001). Recently, MoS_2_ was also demonstrated to have many promising applications in the biomedical field. For instance, it was reported that MoS_2_ materials can be used for photothermal therapy in cancer treatment because of their strong near-infrared absorption feature (Yin et al., 2014; Wang et al., 2015). The MoS_2_ nanosheets with unique direct band gap has been explored for protein and DNA detections (Zhu et al., 2013; Wang et al., 2014). Also, MoS_2_ is an attractive contrast agent used in X-ray computed tomography imaging due to the strong absorbance of X-ray by Mo atoms (Yin et al., 2014). Moreover, because of the weak interaction between the adjacent layers and high surface areas, MoS_2_ can be used as a nano-delivery carrier by surface engineering techniques (Liu et al., 2015).

As one of the most promising applications, the functionalized MoS_2_ nanosheets were demonstrated to have strong inhibiting effects and bactericidal activities against Gram-positive and Gram-negative ESKAPE pathogens (Jack et al., 2013) by destroying their cell membranes (Pandit et al., 2016). Also, The MoS_2_ nanosheets showed the inhibitory activities on the *Escherichia coli* (*E. coli*) due to membrane and oxidative stress. (Yang et al., 2014). In addition, MoS_2_ could also destroy the cell membranes of *E. coli* and extract its phospholipids from the membranes (Wu et al., 2018). Overall, the potential nanomedicine as antibacterial agents for the MoS_2_ nanosheets, including the possible nanotoxicity, is heavily related to their strong interactions with cell membranes. Thus, the study of interactions between the MoS_2_ nanosheets and cell membranes under different conditions and the resultant effects are appealing.

It is noted that the freshly exfoliated MoS_2_ nanosheets (fresh MoS_2_) can absorb hydrocarbon contaminants when they are left in the air and lead to surface aging (named as aged MoS_2_). The aging process could change the surface properties, including hydrophobicity and topology, and then affects the interactions of MoS_2_ with the surroundings, which is mainly characterized by the water contact angles (WCA) on the surface. As an important surface property, WCA affects various functions of many materials, such as catalytic activity (Li et al., 2016), anti-fouling properties (Bano et al., 2015), and water-dispersible (Huang et al., 2018). Many studies have been done to determine the WCAs on the MoS_2_ surfaces (Gaur et al., 2014; Kozbial et al., 2015; Gurarslan et al., 2016). For example, An aging process can increase the WCA on the surface of fresh MoS_2_ from 69.0° ± 3.8° to about 90° after being left in the air for one day, caused by hydrocarbon contaminations (Kozbial et al., 2015). In fact, the WCA of MoS_2_ surface depends on its aging degree. However, how the surface aging process and the resultant change in WCA of a MoS_2_ nanosheet affect its interaction with biomolecules such as the lipid membranes at the bio-nano interface, is yet unclear.

In this paper, we employed molecular dynamics (MD) simulations to study how the surface aging process of MoS_2_ affects its interactions with the lipid membranes of different components, e.g., 1-1-palmitoyl-2-oleoylphosphatidylethanolamine (POPE) and 1-palmitoyl-2-oleoylphosphatidylcholine (POPC). We found that both the MoS_2_ nanosheets (fresh *vs.* aged) can insert into the lipid membranes, which is dominated by the inter-molecules van der Waals interactions. Thus, the interactions from the fresh and aged MoS_2_ nanosheets and the resultant insertion processes were compared. Relative to the aged one, the fresh MoS_2_ nanosheet shows significantly more robust interactions with the membranes and has a deeper insertion depth, regardless of the lipid components. Additionally, free energy changes along the insertion processes were also calculated through the umbrella sampling technique for the two types of MoS_2_ nanosheets. Moreover, the damages to the structure of the lipid membranes caused by the insertions of the fresh and aged MoS_2_ nanosheets were analyzed. Our results provide useful insights for the design of novel MoS_2_-based nanodevices with advanced surface properties.

## Simulation Systems and Methods

### Simulation System

Two types of cell membranes contained different lipid components were modeled here. The first type is modeled with POPE lipid molecules, which are the main component of *E. coli* membranes (Tu et al., 2013). The other type is modeled with POPC lipid molecules, which are quite common in other types of cells (Loschwitz et al., 2020). Those two types of membranes with surface dimensions of 10 × 10 nm^2^ were generated by using CHARMM-GUI (Jo et al., 2009) (http://www.charmm-gui.org), including 316 POPE and 316 POPC lipid molecules, respectively. Subsequently, the membranes were solvated and ionized with 0.15 M NaCl solution and then equilibrated for 100 ns at 300 K and 1 bar. The final membrane structures were used to study their interactions with the MoS_2_ nanosheets.

**FIGURE 1:**
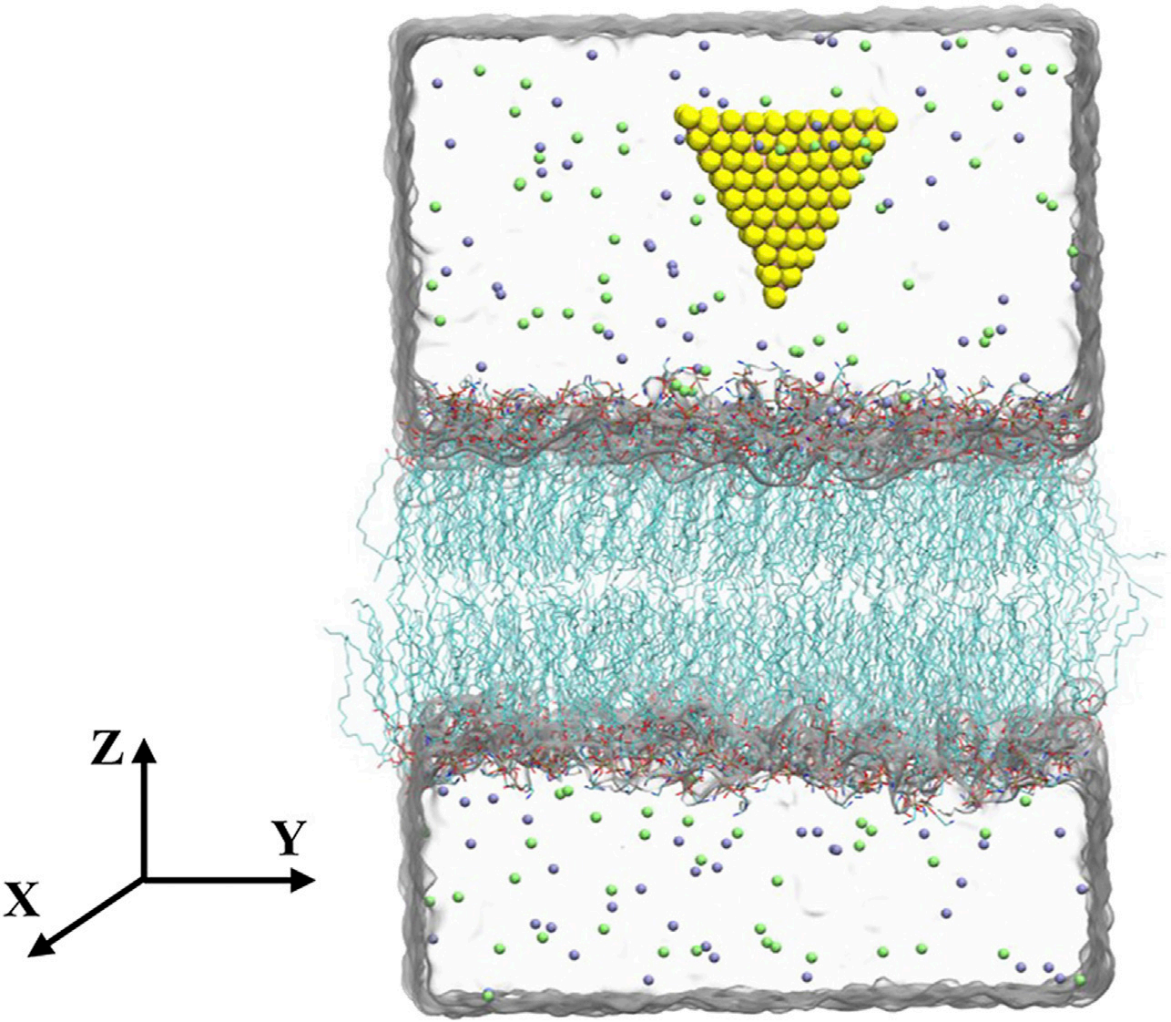
Initial configuration of the simulation system. Molybdenum and sulfur atoms are shown as pink and yellow spheres, respectively. The lipids are in the licorice representation. The carbon atoms are shown in cyan, oxygen atoms in red, phosphorus atoms in blue, nitrogen atoms in brown. Water is shown transparently for clarity. Sodium and chlorine ions are displayed by green and purple spheres, respectively.

The equilateral triangular MoS_2_ nanosheet (side length of 2.89 nm) was modeled by VMD software (Humphrey et al., 1996). This small-sized triangular MoS_2_ nanosheet has been showed that it is capable of penetrating into the bilayer (Gu et al., 2019). For convenience, both the fresh and aged MoS_2_ nanosheets were initially placed above the lipid membranes (POPE and POPC membranes) with the distance from the center of MoS_2_ to the membrane is about 4.2 nm (see Figure 1). In addition, different initial configurations with the bottom edge of MoS_2_ parallel to the lipid membranes were also considered in Supplementary Figure S1A. The simulation box was set to 10 × 10 × 11.68 nm^3^, containing 10,473 water molecules, 48 Na ions and 48 Cl ions.

### Simulation Details

NPT ensembles were employed to simulate the systems. The temperature T = 300 K was maintained by using the velocity-rescaled Berendsen thermostat (Bussi et al., 2007). The pressure (1 bar) was controlled using semi-isotropic Parrinello-Rahman pressostat (Parrinello and Rahman, 1981). The energy parameters for the fresh and aged MoS_2_ nanosheets were adopted from previous works (Table 1) (Luan and Zhou, 2016; Zhang et al., 2019). For water molecule, the TIP3P model was chosen (Jorgensen et al., 1983). The CHARMM 36 force field (Brooks et al., 1983; Mackerell et al., 2004; Klauda et al., 2010) was used to model the lipids and water molecules. The periodic boundary conditions were applied in all three directions (Dolan et al., 2002). The long-range electrostatic interactions were treated with the PME method (Darden et al., 1993) and the van der Waals (vdW) interactions were truncated with a cutoff distance of 1.2 nm. The LINCS algorithm was adopted to constrain the bond vibrations involving hydrogen atoms (Hess et al., 1997), allowing a time step of 2 fs. All the MD simulations were carried out using the GROMACS 5.1.4 package (Hess et al., 2008). Five independent simulations were performed for 200 ns for each system. The snapshots were made by the visual molecular dynamics (VMD) program (Humphrey et al., 1996).

**TABLE 1:** Experimentally determined WCAs (Kozbial et al., 2015) and the resultant energy parameters from theoretical calculations (Luan and Zhou 2016; Zhang et al., 2019) for the fresh and aged MoS_2_ nanosheets, respectively. *σ* and *&epsi;* are the parameters for the vdW interactions in a CHARMM-like form.

Name	WCA	Atom	σ(nm)	ε(kJ/mol)	Charge(e)
Fresh MoS_2_	$∼69^{{}^{\circ}}$	Mo	0.2551	0.5441	0.76
		S	0.3550	1.6744	−0.38
Aged MoS_2_	$∼90^{{}^{\circ}}$	Mo	0.2551	0.5441	0.76
		S	0.3550	1.0450	−0.38

### Calculation of Free Energy Change of Insertion Process

To describe the free energy changes for the insertions of fresh and aged MoS_2_ into the lipid membranes, the potential of mean force (PMF) along the Z-direction, which is perpendicular to the membrane surface, were calculated using umbrella sampling simulations (Torrie and Valleau, 1977; Kumar et al., 1995; Roux 1995). In detail, for a sampling window with a reference distance d_0_, the MoS_2_ nanosheet located at distance d (defined as the distance from the center of MoS_2_ to the membrane) was restrained with a harmonic force

$$\text{F }=\;k\text{ ×}\left(\text{d}-{\text{d}}_{\text{0}}\right)$$ (1)

where *k* = 2,000 kJ mol^−1^ nm^−2^ is the force constant. The spacing of the sampling windows was 0.1 nm and there were 50 windows used in total. For each simulation window, the system was equilibrated for 100 ns, followed by a 10 ns productive run. The PMF curves were obtained by the weighted histogram analysis method (Kirkwood 1935; Efron 1979; Hub et al., 2010).

### Calculation of Orientation Order of Lipid Tails

Insertions of the MoS_2_ nanosheets into the lipid membranes can induce mechanical deformations of lipid orientations and destroy the integrity of lipid membranes. To some extent, those destructs can be indicated by the orientation order of the lipid tails, S_chain_, written as:

$${\text{S}}_{\text{chain}}\;=\text{ 0}{\text{.}5\text{ < }3\text{cos}}^{2}\theta -1\text{ >}$$ (2)

here *θ* is the angle between the bilayer normal and the geometrical arrangement of the hydrocarbon chain, which is defined as the vector between the first and last carbon atom (**see** Supplementary Figure S2). The values of S_chain_ = 1, 0.5, and 0 represent perfect alignment, antialignment, and random orientation, respectively (Fu et al., 2020).

## Results And Discussion

### Derivation of Energy Parameters for the MoS_2_ Nanosheets

The adsorption of hydrocarbon contaminations during aging process changes the surface properties and affects the interactions between the MoS_2_ nanosheets and the surroundings, which can be mainly characterized by the water contact angles (WCAs) on the surface. As one of the most important surface properties, the WCA is directly associated with the surface hydrophobicity/hydrophilicity, which is influenced by the adsorption of hydrocarbon contaminations and the possible changes of the termination groups at the outermost MoS_2_ along the aging process. Actually, a variety of water-MoS_2_ contact angles have been experimentally determined in the literatures and it was found that the WCA closely depends on the surface aging conditions of MoS_2_ and/or substrate materials (Gaur et al., 2014; Kozbial et al., 2015; Gurarslan et al., 2016). In previous studies (Govind Rajan et al., 2016; Leroy 2016; Heiranian et al., 2017; Sresht et al., 2017; Khalkhali et al., 2018), the WCA is always served as the dominated quantity to extract energy parameters of novel 2D nanomaterials, including the MoS_2_ nanosheets here. For instance, through theoretical analysis and simulation, recent studies (Luan and Zhou, 2016; Zhang et al., 2019) have successfully reproduced the experimentally determined WCAs of the MoS_2_ nanosheets under different conditions (Gaur et al., 2014; Kozbial et al., 2015; Gurarslan et al., 2016) by only modifying the Lennard-Jones parameter *ε*_*S*_ (the depth of the potential well of a sulfur atom) and observed an excellent linear relationship between the *ε*_*S*_ and WCAs. For convenience, we here directly used these deduced energy parameters to explore how the aging process affects the interactions of the MoS_2_ nanosheets with the lipid membranes. The related WCAs determined by experiments (Kozbial et al., 2015) and the resultant energy parameters from the aforementioned theoretical studies (Luan and Zhou, 2016; Zhang et al., 2019) for the fresh and aged MoS_2_ surfaces are given in Table 1. We expect these energy parameters could sufficiently describe the interactions between the fresh/aged MoS_2_ nanosheet and the lipid membranes.

### Insertion of the Fresh and Aged MoS_2_ Nanosheets Into the Lipid Membranes

**FIGURE 2:**
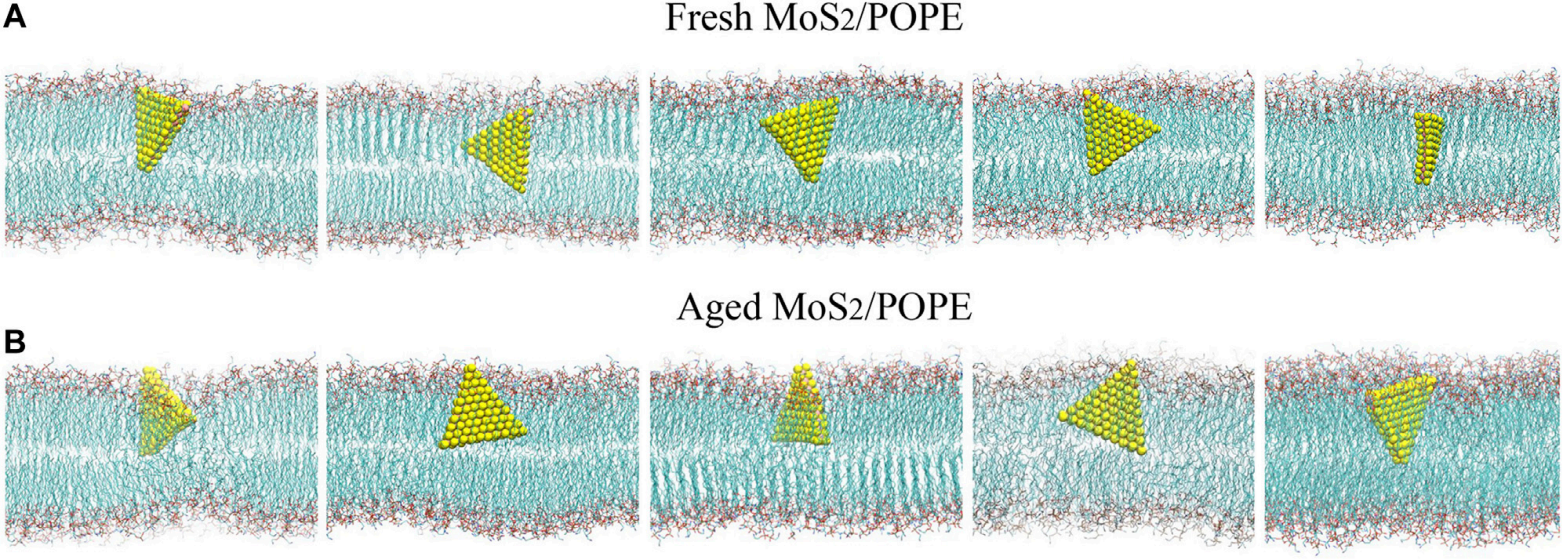
MD simulations indicated both the **(A)** fresh and **(B)** aged MoS_2_ nanosheets can insert into the POPE lipid membrane. Final conformations of the MoS_2_ nanosheets/lipid membrane from five independent simulation runs are shown.

We first studied the interactions between MoS_2_ and the POPE lipid membrane. The initial conformation of the simulation system was illustrated in Figure 1, wherein the distance from the center of MoS_2_ to the membrane is about 4.2 nm with one of the vertexes pointing toward the membrane (details can be found in the Methods section). Figure 2 showed the final conformations of the two types of MoS_2_ nanosheets (fresh *vs.* aged) from five independent MD simulations. We found that both MoS_2_ (fresh *vs.* aged) could insert into the lipid membrane and were finally capsulated by the lipids with their geometrical arrangements perpendicular to the bilayer surfaces. In addition, the capsulated MoS_2_ (fresh *vs.* aged) were mostly in contact with the hydrocarbon chains of the lipids and parallel to the chains. It should be noted that the insertion of the MoS_2_ nanosheet was independent of its initial orientation with respect to the lipid membranes (Supplementary Figure S1). The results revealed that both the MoS_2_ nanosheets (fresh *vs.* aged) can insert into the lipid bilayer of POPE membrane, independent of their surface aging process. Similarly, both the two types of MoS_2_ nanosheets (fresh *vs.* aged) can penetrate into the POPC lipid bilayer membranes (Supplementary Figure S1 and S3).

### Analysis of the Insertion Processes of the MoS_2_ Nanosheets

**FIGURE 3:**
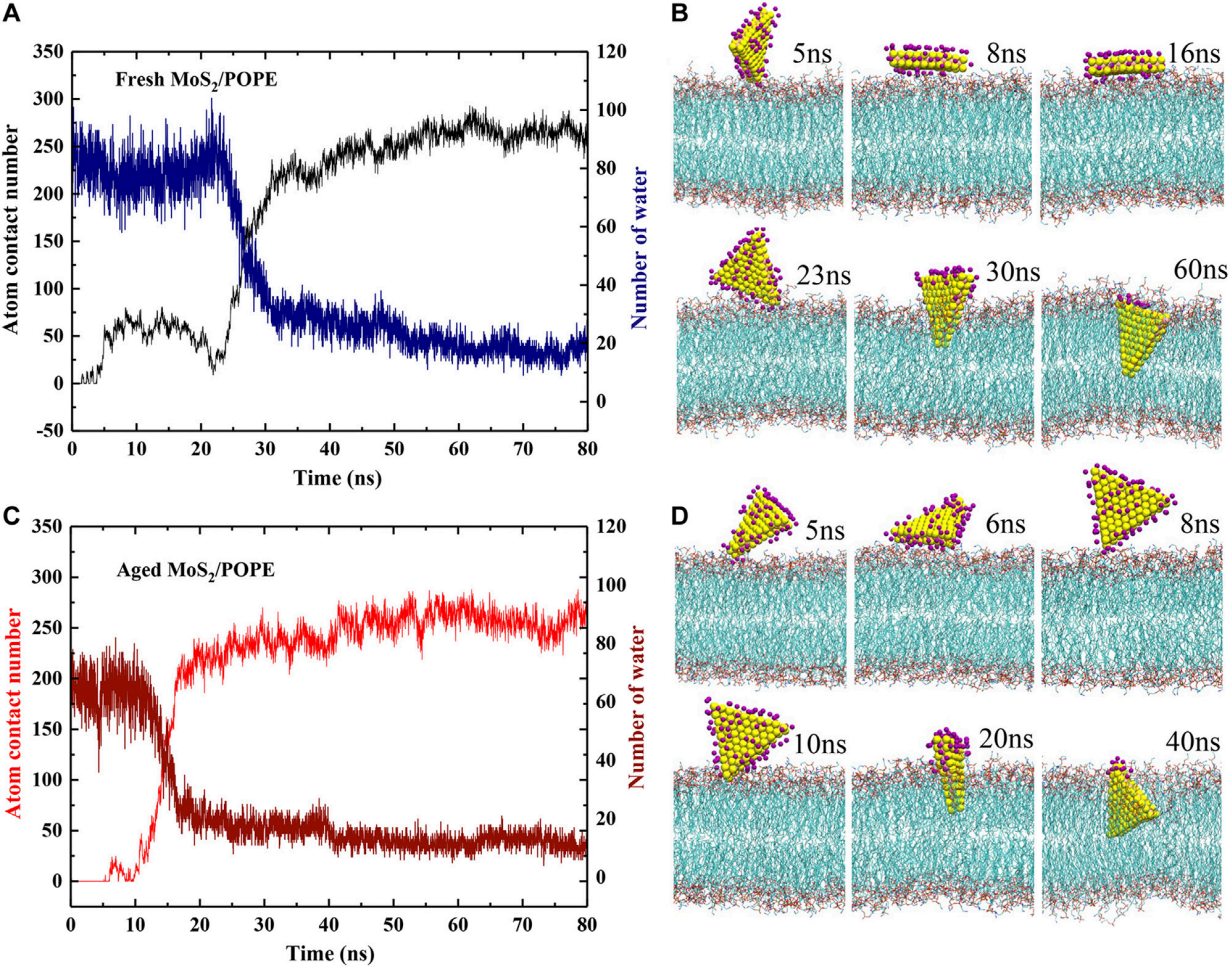
Insertion processes of the MoS_2_ nanosheets into the POPE lipid membrane. **(A, C)** The time-dependent atom contact numbers and surface water molecules for the **(A)** fresh and **(C)** aged MoS_2_ nanosheets. **(B, D)** The typical conformations of the **(B)** fresh and **(D)** aged MoS_2_ nanosheets during the insertion processes. The dark purple spheres represent the water oxygen atoms.

In order to better understand the above insertion processes, the time-dependent atom contact numbers between the MoS_2_ nanosheets (fresh and aged surfaces) and the lipid membrane from two representative MD simulation trajectories were further analyzed (see Figures 3A,C). Here, the MoS_2_ nanosheet and any heavy atoms of a lipid are considered to be in contact if the distance between them is less than 5 Å. In addition, the typical conformations of the MoS_2_ nanosheets (fresh *vs.* aged) relative to the POPE membrane at four key time points were also showed in Figures 3B,D. Initially, as for the fresh MoS_2_ nanosheet, it was freely moved in the water, and has intermittent contact with the lipid molecules, yielding the contact numbers less than 25 (0∼5 ns). At t = 5 ns, fresh MoS_2_ began to contact the surface, with a point-to-face orientation targeting to the membrane. At t = 8 ns, MoS_2_ changed its binding conformation to a face-to-face orientation, with an increase in the contact numbers close to 75. From t = 8 ns to t = 16 ns, fresh MoS_2_ adhered to the lipid membrane in a face-to-face manner, and the atom contact numbers keep nearly constant value (∼75). At t = 23 ns, the fresh MoS_2_ nanosheet was tilted with its vertex penetrating into the membrane, along with a slightly decrease in the contact numbers compared to that at t = 16 ns. Starting from t = 23 ns, the MoS_2_ nanosheet began to insert into the lipid membrane, with a significant increase in the contact numbers. At t = 30 ns, half part of the MoS_2_ nanosheet was buried in the lipid membrane and the atom contact numbers increased to ∼225. After t = 30 ns, we noticed that the atom contact numbers were only slowly increased, suggesting that the speed of further insertion of the MoS_2_ nanosheet has slowed down. From t = 60 ns, the interactions of fresh MoS_2_ with the POPE lipid membrane reached a saturated state, with the limited fluctuations in the contact numbers (∼275). As for the aged MoS_2_ nanosheet, the changes of the contact numbers and binding conformations are comparative to the aforementioned fresh MoS_2_ nanosheet, as illustrated in Figures 3C,D. One should note that the insertion processes of the MoS_2_ nanosheets are somewhat stochastic. In some cases, the simulation trajectories (Supplementay Figure S4) show that the MoS_2_ nanosheets (fresh vs. aged) can directly insert into the membrane without undergoing the preorganization of its conformation on the membrane. In other words, since the orientation of MoS_2_ nanosheet relative to membrane is random when it approaches the membrane, preorganization of MoS_2_ nanosheet conformation before insertion is sometimes needed to facilitate the insertion process. Then the fluctuation of the atom contact numbers between the MoS_2_ nanosheet and the lipid membrane was observed, as that shown in Figure 3A during simulation time t = 5∼23 ns. In the previous studies, we also observed the similar process that the adsorption of MoS_2_ on the membrane surface could adjust its conformation (Gu et al., 2019). But sometimes, if the approached conformation is suitable for insertion in chance, the preorganization process is absent, as that shown in Figure 3C during simulation time t = 8∼20 ns and Supplementary Figure S4. Moreover, though the insertion rate of the fresh MoS_2_ nanosheet into the lipid membrane shown in Figure 3A seems like not as good as that of the aged MoS_2_ nanosheet (see Figure 3C), the average insertion time over the five simulation trajectories for fresh MoS_2_ (about 40 ns) is shorter than that of the aged one (around 52 ns), indicating that the average insertion rate of fresh MoS_2_ is faster.

Moreover, we also explored the dehydration of both MoS_2_ (fresh *vs.* aged) during entering the lipid membrane by calculating the number of water molecules binding to their surfaces (Figures 3A,C). Here, a binding water molecule was considered only if its oxygen atom is within 3.5 Å from the MoS_2_ surface (defined in Supplementary Figure S5B). Initially, in the case of fresh MoS_2_, the number of water molecules on its surface is about 85. From t = 5 ns to t = 23 ns, the number of water molecules fluctuated between 70 and 85 as the unstable contact of MoS_2_ with the lipid membrane. After t = 23 ns, the number of water molecules decreased sharply, suggesting that the interaction of MoS_2_ and phospholipids can quickly overcome the barrier caused by water molecules in the dehydration process. As the insertion process proceeded (t > 30 ns), the dehydration process of MoS_2_ started to slow down. At t = 60 ns, the fresh MoS_2_ nanosheet completely inserted into the lipid membrane and only the part close to the phospholipid headgroups was in contact with water molecules (∼20). For the aged MoS_2_ nanosheet, its dehydration process was similar to fresh MoS_2_ (Figures 3C,D). At the beginning (from t = 0 ns to t = 5 ns), the number of water molecules on the fresh MoS_2_ surface is about 20 more than that of the aged one (Supplementary Figure S5C), indicating that the fresh MoS_2_ nanosheet is indeed more hydrophilic. However, at the end of insertion, the number of water molecules left on the MoS_2_ nanosheets is controlled by both the insertion depth and the conformation of the inserted MoS_2_, where the latter is some of random in simulations. As shown in Supplementary Figure S6, when the inserted conformation is similar, the fresh MoS_2_ nanosheet has fewer water molecules left on its surface due to its larger insertion depth than the aged counterpart.

### Interaction Energies Between the MoS_2_ Nanosheets and the Lipid Membranes

**FIGURE 4:**
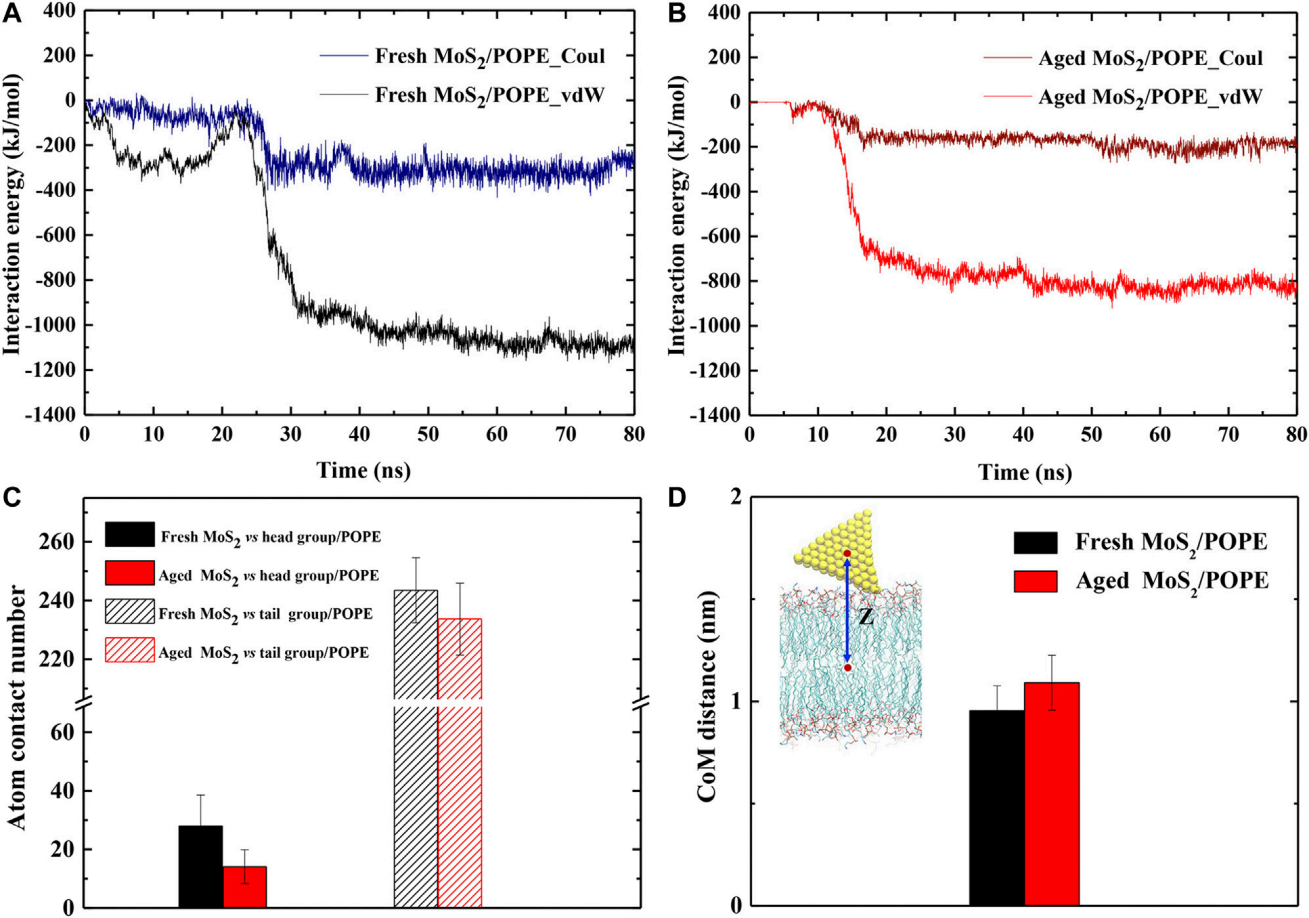
Surface aging affects the interactions of MoS_2_ with the lipid membranes. The time-dependent interaction energies (vdW and Coul energies) between the **(A)** fresh and **(B)** aged MoS_2_ nanosheets and the POPE lipid membrane during the insertion processes. **(C)** The average atom contact numbers between the fresh/aged MoS_2_ nanosheets and the head/tail groups of POPE lipid molecules. **(D)** The average center of mass (CoM) distances between the fresh/aged MoS_2_ nanosheets and the POPE lipid membrane.

To further characterize how the aging of surface affects the interaction of MoS_2_ with its surroundings, we calculated the time evolution of the inter-molecule interaction energies between both MoS_2_ (fresh *vs.* aged) and the lipid membranes. Figures 4A,B showed the time evolutions of vdW and Coulombic (Coul) interaction energies between the fresh/aged MoS_2_ nanosheets and the POPE lipid membrane from the above representative trajectories. As the insertion of MoS_2_ proceeded, the inter-molecule interaction energies (including vdW and Coul energies) were gradually lowering. When the insertion process finished, it was found that the vdW energies were much stronger than the Coul energies for both the two types of MoS_2_ nanosheets. More specifically, the vdW energy (∼−1,140 kJ/mol) was ∼3 times larger than the Coul energy (∼−390 kJ/mol) for the fresh MoS_2_ nanosheet, and the vdW energy (∼−850 kJ/mol) was ∼3 times larger than the Coul energy (∼−260 kJ/mol) for the aged MoS_2_ nanosheet. Therefore, the vdW interactions, or hydrophobic interactions to some extent, drive the insertion of the MoS_2_ nanosheets into the lipid membrane. Moreover, we have calculated the interaction energies by averaging the last 10 ns over all independent trajectories (Supplementary Figure S7A). It should be noted that all the Coul, vdW, and total energies between the fresh MoS_2_ nanosheet and the membrane were more robust than those of the aged counterpart.

Generally, the Coul interactions mainly arise from the contact of MoS_2_ with the polar head regions of lipid molecules (the negatively charged phosphate groups PO_4_^−^ and the positively charged amino groups NH_3_^+^); while the vdW interactions mainly result from the contact of MoS_2_ with the hydrophobic tail region of the lipid chains. Hence, we further analyzed the contact of the MoS_2_ nanosheets with these two regions respectively (Figure 4C). The average contact numbers of the fresh MoS_2_ nanosheet with the head groups (25) and tail groups (245) were larger than that in the case of the aged nanosheet (17, 230), which is consistent with the trend of vdW and Coul interaction energies for these MoS_2_ nanosheets (Supplementary Figure S7A). Moreover, we noticed that the stable binding positions in the membrane for the fresh and aged MoS_2_ nanosheets are different. Thus, we also calculated the averaged center of mass (CoM) distances between the MoS_2_ nanosheet and the membrane along the Z-axis and the results were given in Figure 4D. The depth of the fresh MoS_2_ nanosheet entering the lipid membrane was greater than that of the aged counterpart. This slight difference in the insertion depths is also reflected in the difference of their vdW interactions which are mainly from the contact with lipid tails. Similarly, we analyzed the interaction energies and CoM distances between the MoS_2_ nanosheets (fresh *vs.* aged) and the POPC lipid membrane, and also confirmed that fresh MoS_2_ has a stronger interaction with the membrane and exhibited greater insertion depth (Supplementary Figures S7B, S8).

### The Free Energy Changes of the MoS_2_ Nanosheets Insertion

**FIGURE 5:**
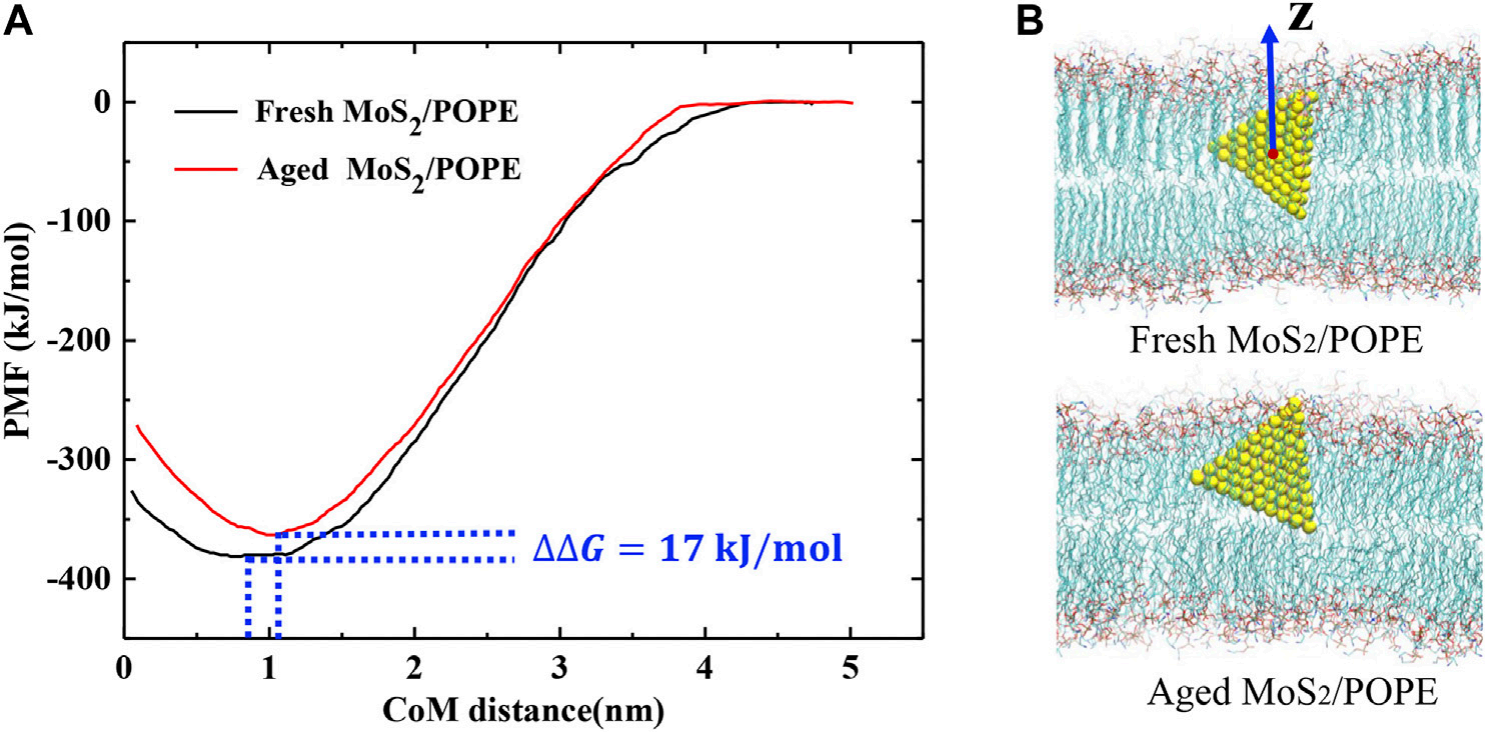
Free energy changes of the MoS_2_ nanosheets insertion into the POPE lipid membrane. **(A)** Potential of mean force (PMF) curves for insertion of the fresh/aged MoS_2_ nanosheets calculated from umbrella sampling simulations. **(B)** The representative conformation of the fresh/aged MoS_2_ nanosheets interacting with the POPE lipid membrane corresponding to the potential wells.

To evaluate the free energy changes during the insertion process, we calculated the potential of mean force (PMF) along the Z-direction, which is perpendicular to the bilayer surfaces, using the umbrella sampling method (details can be seen in the Methods section). Three isolated sets of PMFs were calculated from three independent simulations and they showed similar profiles (Supplementary Figure S9 and Figure 5). One typical PMF profile for insertion of the fresh and aged MoS_2_ nanosheets into the POPE lipid membrane was displayed in Figure 5, respectively. Figure 5A shows that the lowest values on the PMF curves relative to the CoM distance are 0.8 and 1 nm for fresh and aged MoS_2_, respectively, in agreement with the results in Figure 4D. The PMF calculation also indicated that the insertion of fresh MoS_2_ was ∼17 kJ/mol higher than the aged one. Therefore, the fresh MoS_2_ nanosheet was more energetically favorable to insert into the lipid bilayer than the aged one, though both MoS_2_ (fresh *vs.* aged) exhibited the capability of penetrating into the POPE membrane.

### The Impact of the MoS_2_ Nanosheets Insertion on Membrane Properties

**FIGURE 6:**
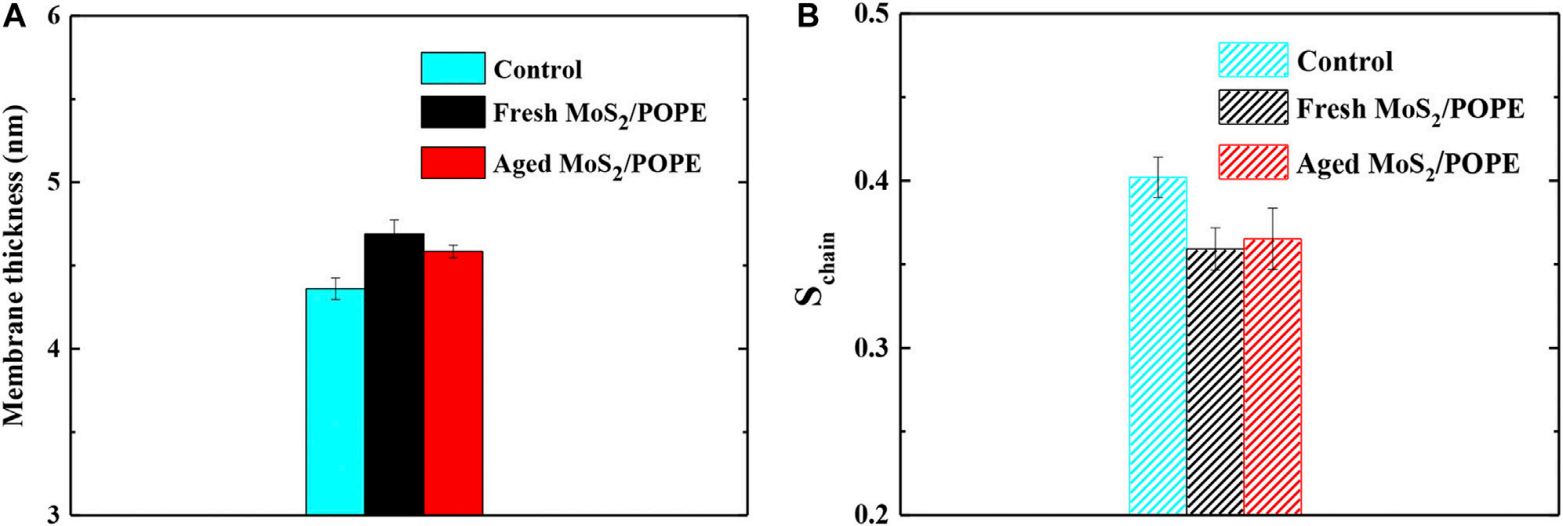
Insertions of the fresh/aged MoS_2_ nanosheet cause structural damage to the POPE lipid membrane. **(A)** The average thickness and **(B)** lipid tail order of POPE membrane over the last 10ns trajectories from five independent simulations.

Previous studies have demonstrated that the MoS_2_ nanosheets can destroy the cell membranes (Jack et al., 2013; Wu et al., 2018). Here, to explore the effects of insertion of the fresh/aged MoS_2_ nanosheets on the properties of the POPE membrane, we calculated the membrane thickness and lipid tail order in terms of chain order S_chain_ (as defined in Methods) within 10 Å from MoS_2_. Figure 6A showed that the insertion of both MoS_2_ (fresh and aged) can make the membrane thickness larger than that of the control group (without MoS_2_). It was worth noting that the fresh MoS_2_ made the membrane thickness larger than that of aged one, which may be due to the fresh MoS_2_ nanosheet exhibited a greater insertion depth (Figure 4D). In addition, as shown in Figure 6B, both the MoS_2_ nanosheets reduced the lipid tail order, implying that the structure of the POPE lipid membrane has been considerably damaged. Surprisingly, the lipid tail order of fresh MoS_2_ was lower than that of aged MoS_2_, illustrating that fresh MoS_2_ made the lipid membrane more disordered. For the POPC lipid membrane, the thickness and lipid tail order of the membrane were also calculated, and the results were consistent with the POPE membrane (Supplementary Figure S10). These results suggested that the fresh MoS_2_ nanosheet might have more damage to the lipid membranes.

## Conclusion

In this work, we investigated how the surface aging of the MoS_2_ nanosheet affect its interaction with the lipid membranes (POPE and POPC) through all-atom molecular dynamics simulations. Specifically, insertions of the fresh and aged MoS_2_ nanosheets into the lipid membranes contained different components were compared. Our results demonstrate that even though both MoS_2_ (fresh *vs.* aged) can insert into the lipid membranes, the fresh MoS_2_ nanosheet shows significantly stronger interaction with the lipid membranes than the aged one. The deeper insertion of fresh MoS_2_ can be attributed to the stronger van der Waals interaction with lipid molecules. Free energy calculations during the insertion processes further verify that the fresh MoS_2_ nanosheet is more energetically favorable in penetrating into the lipid membranes than the aged one. Furthermore, the insertion of fresh MoS_2_ makes the lipid tail order lower than that of aged MoS_2_, indicating that the former exhibits a stronger damaging effect on the membranes than the latter. Our findings revealed that MoS_2_ is a surface-aging-dependent material in interacting with cell membranes, which may shed light on future applications in biomedicine.

## Data Availability

The original contributions presented in the study are included in the article/Supplementary Material, further inquiries can be directed to the corresponding author.
