# Supplementary material for: Membrane Insertion of MoS2 Nanosheets: Fresh vs. Aged
Source: Front Chem. 2021 Jun 25;9:706917. doi: 10.3389/fchem.2021.706917 (PMC8267466; doi:10.3389/fchem.2021.706917)
Supplement: Supplementary file 1 [file DataSheet1.docx]

**Supporting Information**

**Membrane Insertion of MoS_2_ Nanosheets: Fresh *vs.* Aged**

Rui Ye^1,2^, Wei Song^1,2^, Xinwen Ou^1^, Zonglin Gu^3^, and Dong Zhang^2,4*^

^1^Department of Physics, Zhejiang University, Hangzhou 310027, China

^2^Institute of Quantitative Biology, Zhejiang University, Hangzhou 310027, China

^3^College of Physical Science and Technology, Yangzhou University, Yangzhou, Jiangsu 225009, China

^4^College of Life Sciences, Zhejiang University, Hangzhou 310027, China

*All correspondences should be addressed to: zhangd_iqb@zju.edu.cn


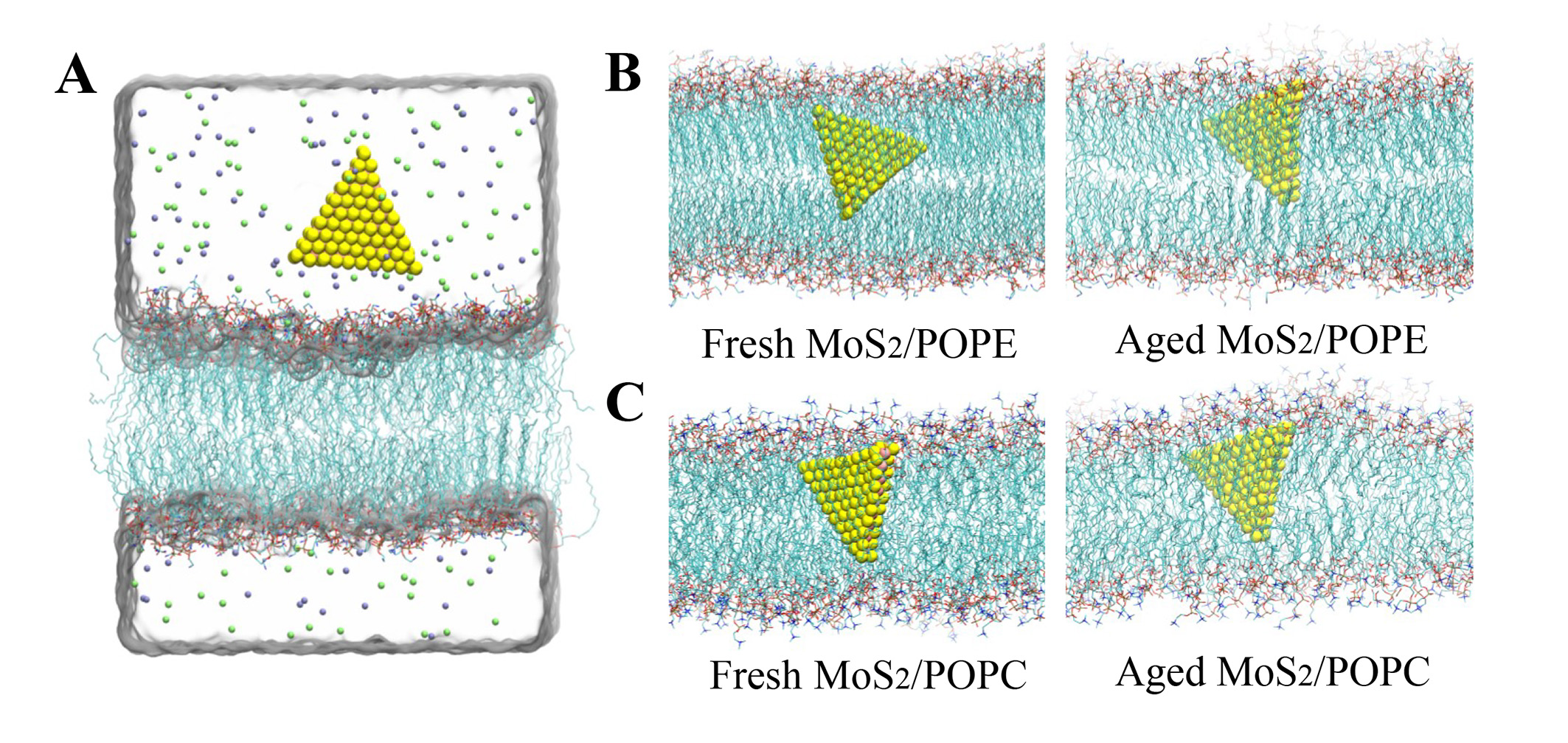


**Figure S1.** (A) Initial configuration of the simulation system. Molybdenum and sulfur atoms are shown as pink and yellow spheres, respectively. The lipids are in the licorice representation. The carbon atoms are shown in cyan, oxygen atoms in red, phosphorus atoms in blue, nitrogen atoms in brown. Water is shown transparently for clarity. Sodium and chlorine ions are displayed by green and purple spheres, respectively. Final conformations of the interaction between the fresh / aged MoS_2_ nanosheets and the POPE (B) / POPC (C) lipid membranes.


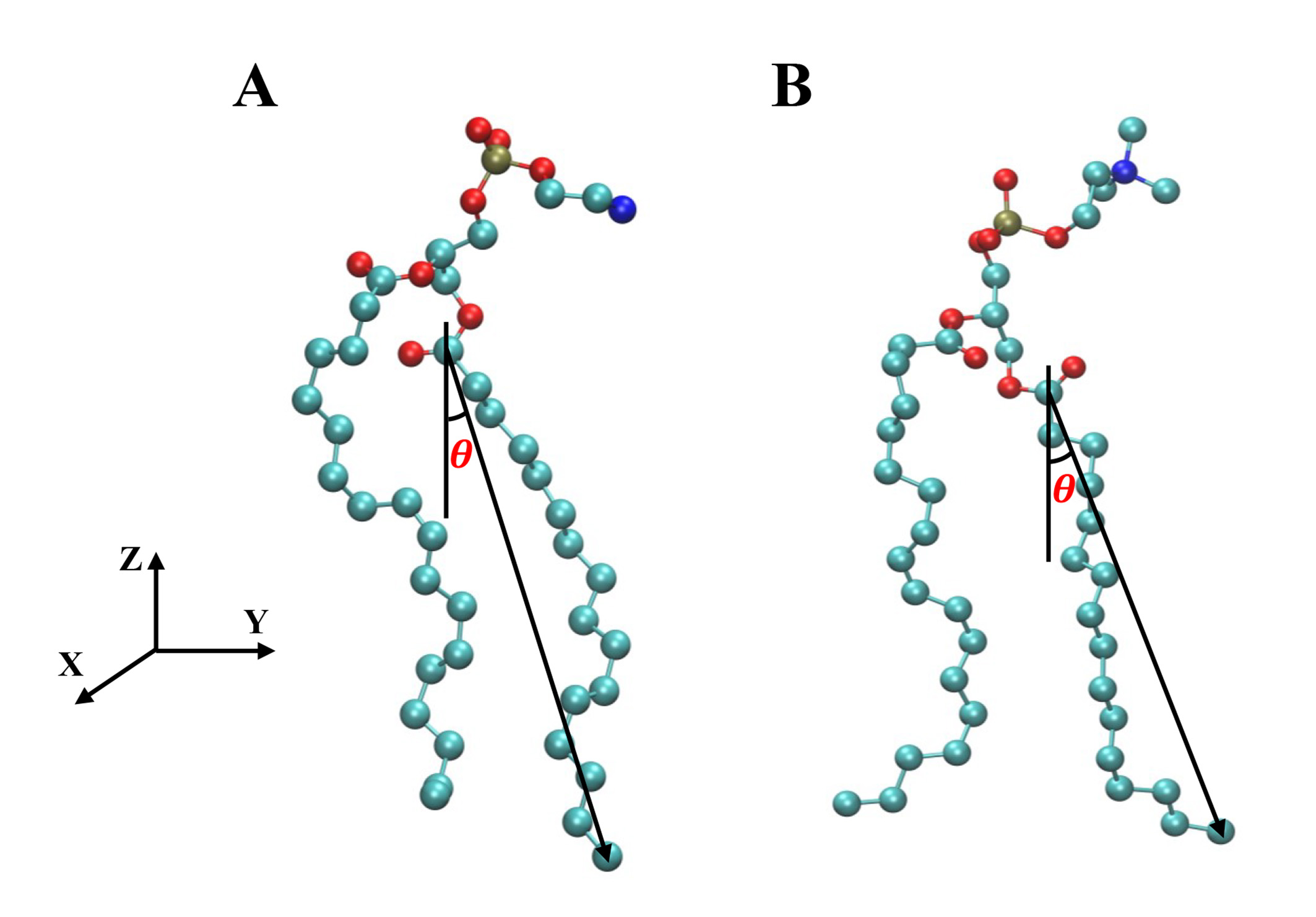


**Figure S2.** Definition of the lipid tail tilt angle of the POPE (A) and POPC (B) lipid membranes. The planar bilayer normal is in the Z-direction.


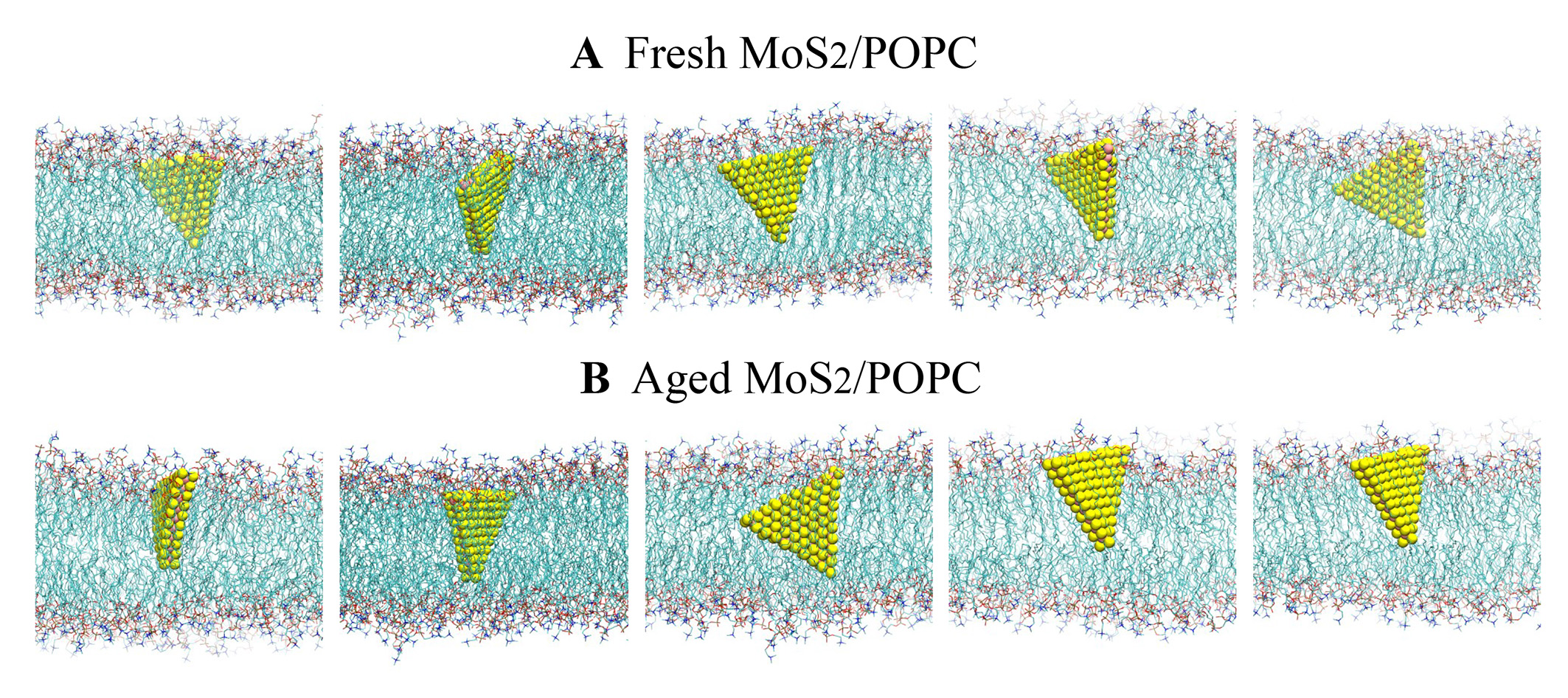


**Figure S3.** MD simulations indicated both the (A) fresh and (B) aged MoS_2_ nanosheets can insert into the POPC lipid membrane. Final conformations of the MoS_2_ nanosheets / lipid membrane from five independent simulation runs are shown.


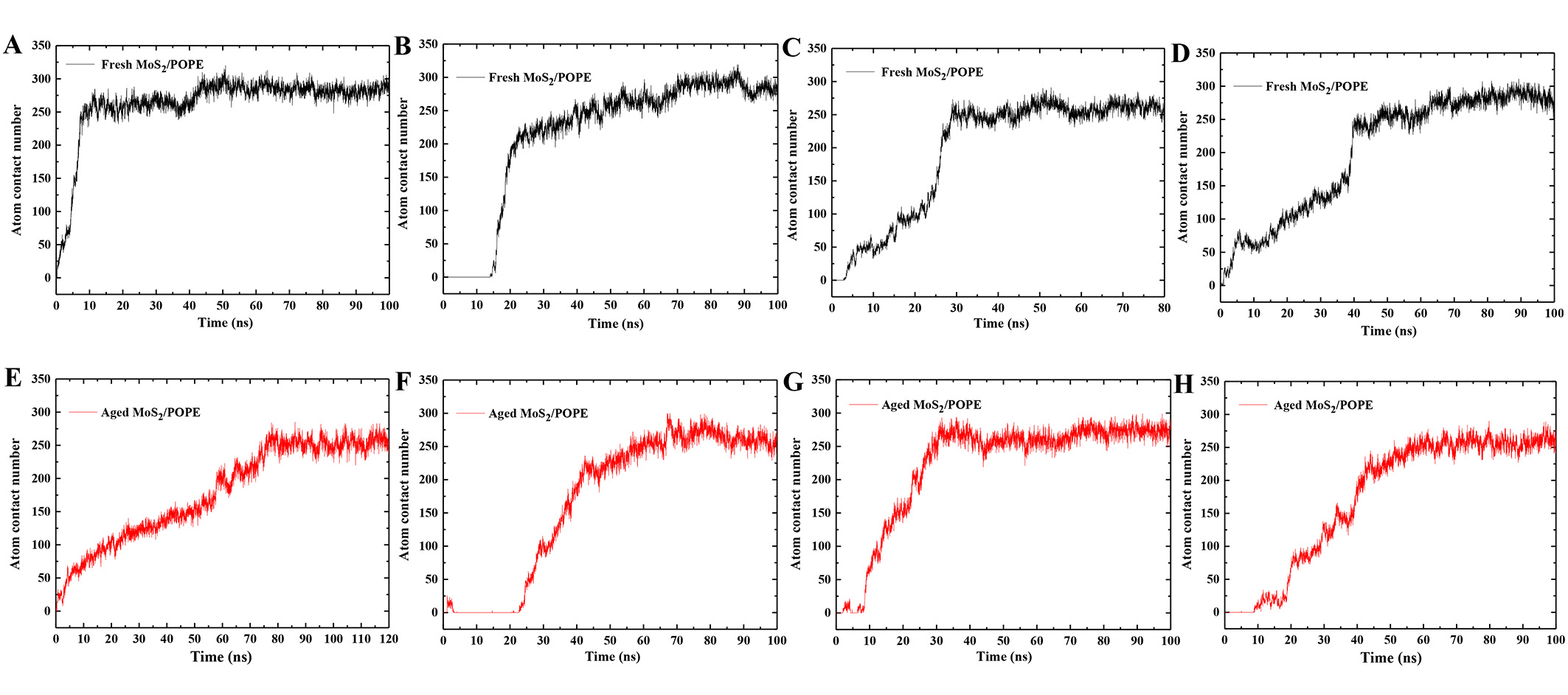


**Figure S4.** Insertion processes of the MoS_2_ nanosheets into the POPE lipid membrane. The time-dependent atom contact numbers of the (A, B, C, D) fresh and (E, F, G, H) aged MoS_2_ nanosheets for other independent simulations.


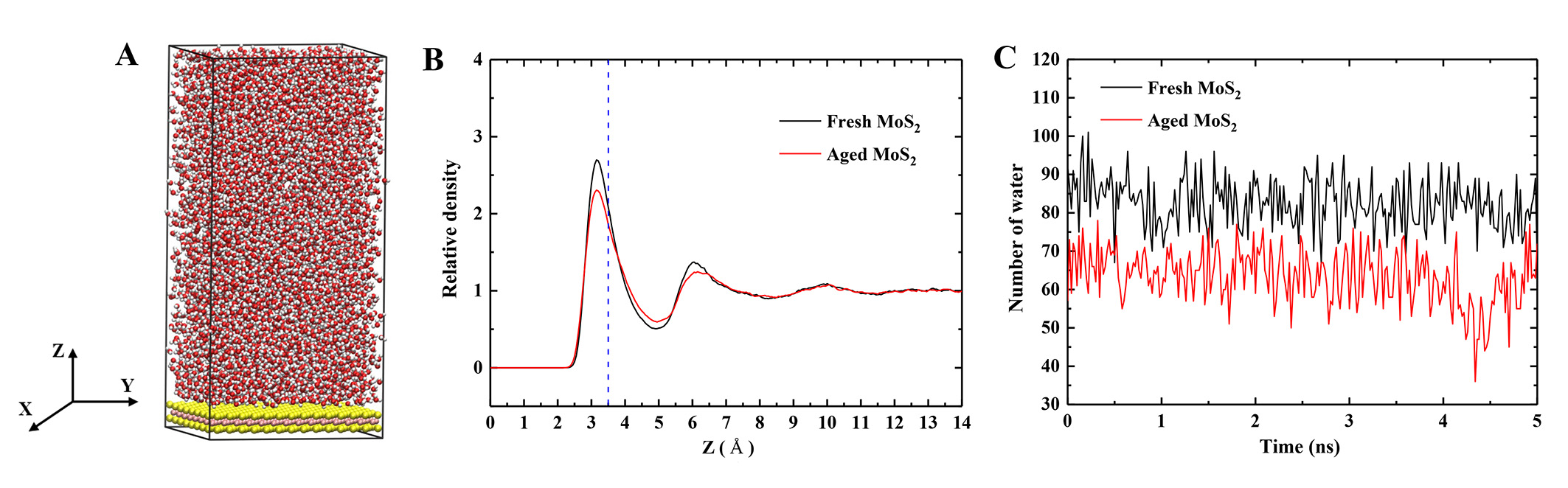


**Figure S5.** (A) Snapshot of MoS_2_ substrate and water. Molybdenum and sulfur atoms are shown as pink and yellow spheres, respectively. The oxygen atoms are represented in red, hydrogen atoms in white. (B) The distribution of the relative density of water on the fresh/aged MoS_2_ substrate surfaces along the Z axis. (C) The number of surface water molecules in the first 5 ns of the fresh/aged MoS_2_ nanosheets from the two representative trajectories in Figure 3.

**
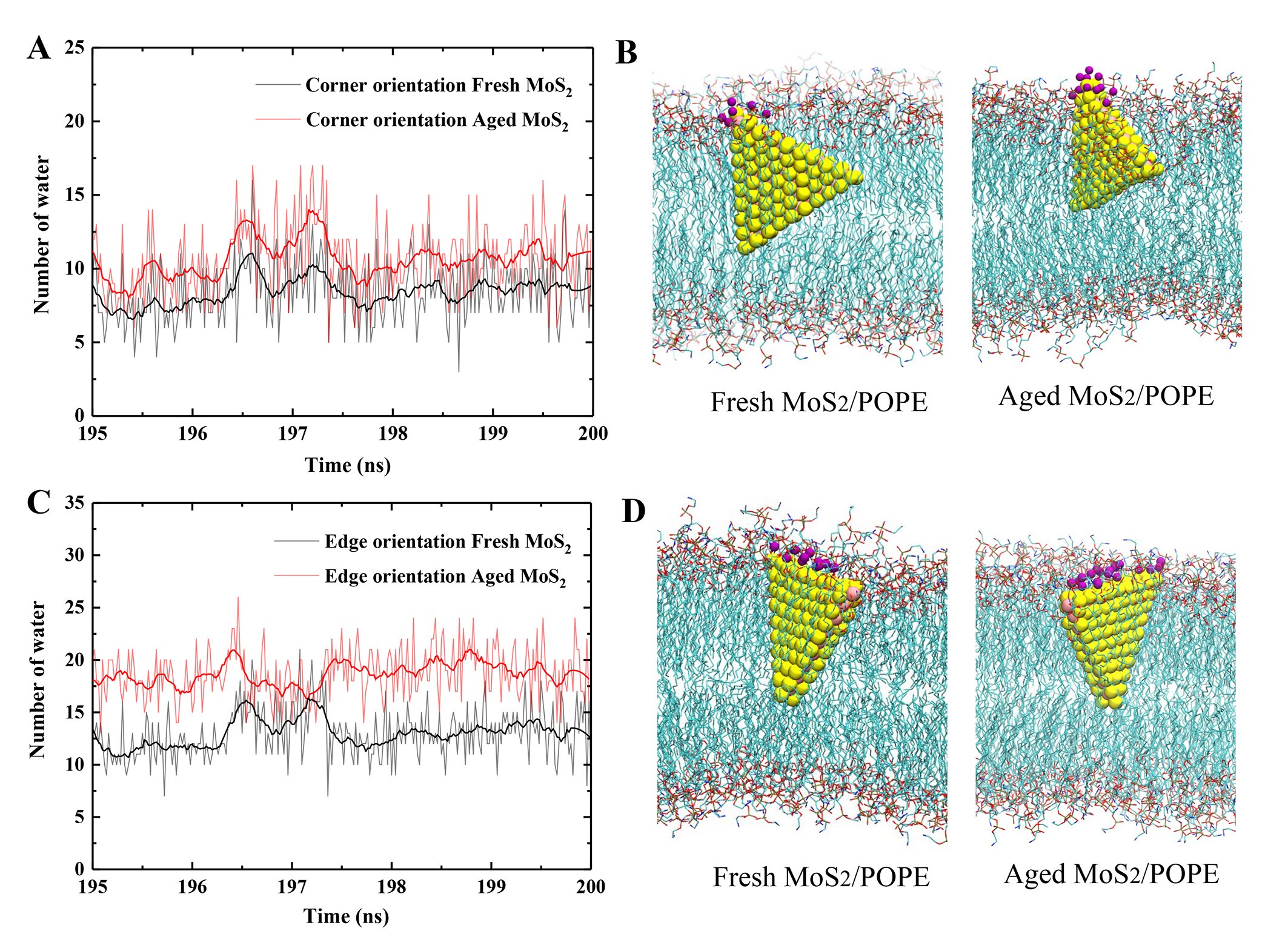
**

**Figure S6.** The number of surface water molecules in the last 5ns and the corresponding final conformations of the simulations for the fresh/aged MoS_2_ nanosheets. For corner (A, B) and edge (C, D) orientation.


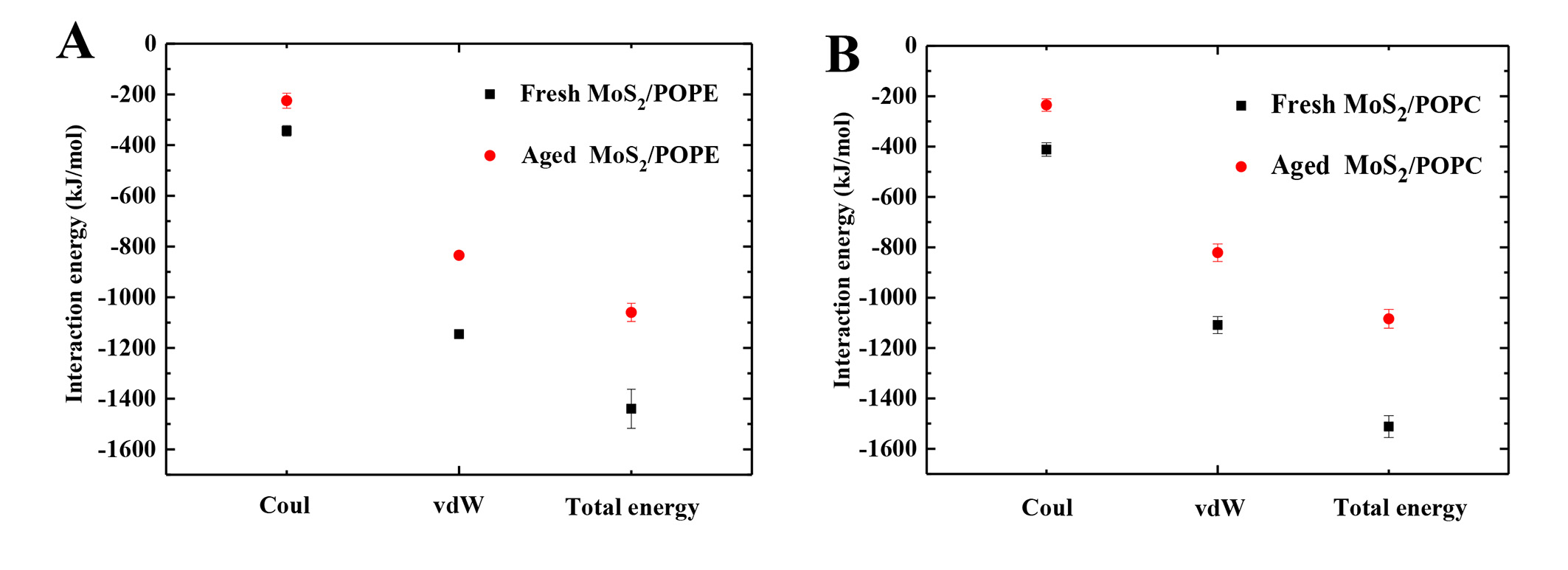


**Figure S7.** Coulombic (Coul), van der Waals (vdW) and total energies between the POPE (A) / POPC (B) lipid membranes and the two types of MoS_2_ nanosheets averaged from the last 10 ns of the simulations out of five independent trajectories.


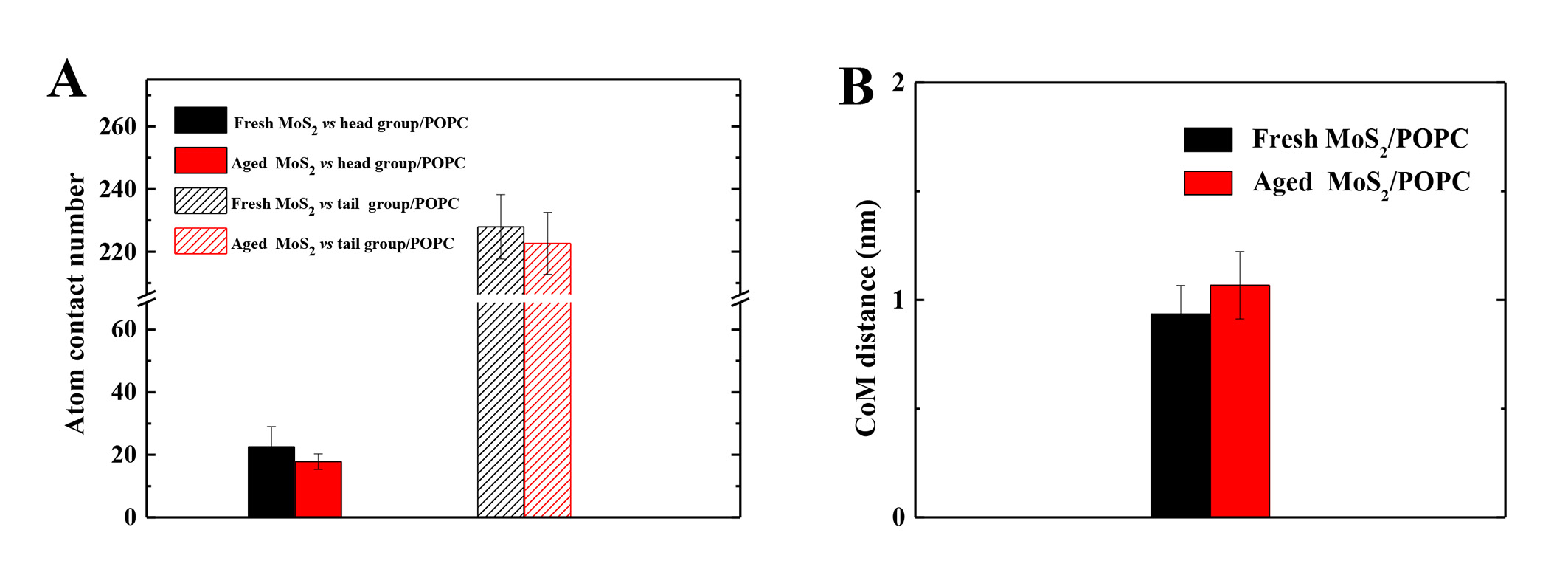


**Figure S8.** (A) The average atom contact numbers between the fresh/aged MoS_2_ nanosheets and the head/tail groups of POPC lipid molecules. (B) The average center of mass (CoM) distances between the fresh/aged MoS_2_ nanosheets and the POPC lipid membrane.


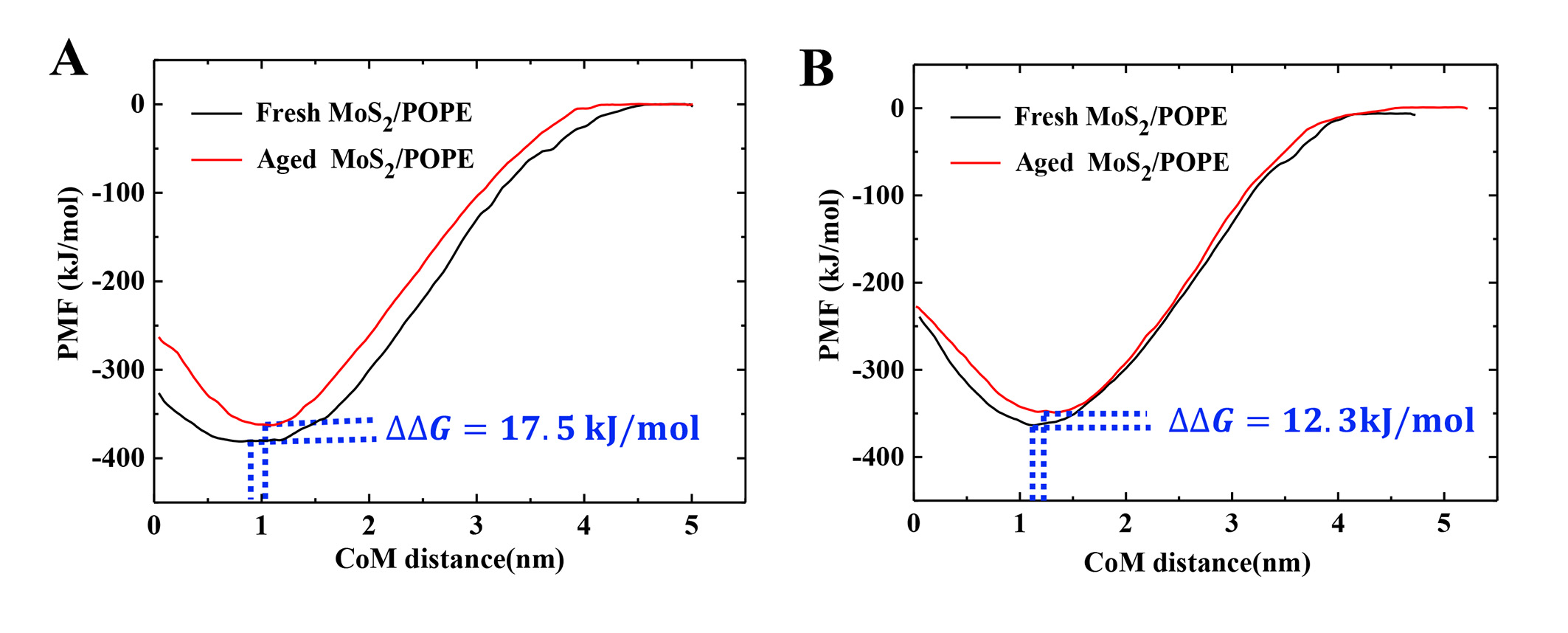


**Figure S9.** Potential of mean force (PMF) curves for other independent simulations.


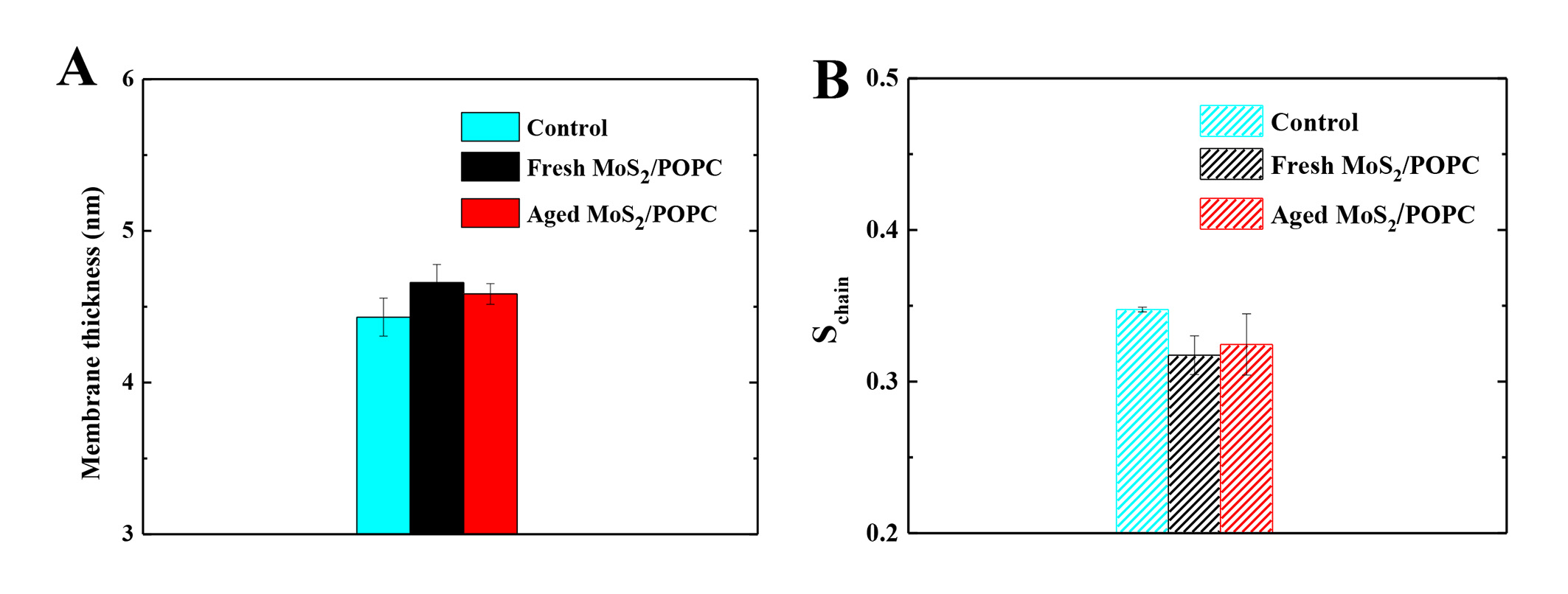


**Figure S10.** (A) The average thickness and (B) lipid tail order of the POPC membrane over the last 10ns trajectories from five independent simulations.
